# Harmonization of MRI sequences across ERN EpiCARE centers

**DOI:** 10.1002/epi4.13115

**Published:** 2025-02-12

**Authors:** Sophie Adler, Felice D'Arco, Kshitij Mankad, Martin Kyncl, Alexis Arzimanoglou, Petr Marusic

**Affiliations:** ^1^ UCL Great Ormond Street Institute of Child Health London UK; ^2^ Collaborating Partner of the ERN EpiCARE Great Ormond Street Hospital London UK; ^3^ Department of Radiology, Second Faculty of Medicine and Motol University Hospital, Member of the ERN EpiCARE Charles University Prague Czech Republic; ^4^ Epilepsy Unit, Child Neurology Department Hospital San Juan de Dios, Member of the ERN EpiCARE Barcelona Spain; ^5^ Department of Neurology, Second Faculty of Medicine and Motol University Hospital, Member of the ERN EpiCARE Charles University Prague Czech Republic

**Keywords:** epilepsy, magnetic resonance imaging, postprocessing

## Abstract

Neuroimaging investigations are fundamental in the diagnosis of patients with epilepsy. The International League Against Epilepsy (ILAE) harmonized neuroimaging of epilepsy structural sequences (HARNESS‐MRI) protocol was intended as a generalizable structural MRI protocol. The European Reference Network for Rare and Complex Epilepsies, EpiCARE, includes 50 centers, across 26 countries, with expertise in epilepsy. We investigated adherence to the HARNESS‐MRI protocol across EpiCARE. A survey on the clinical use of imaging and postprocessing methods in epilepsy patients was distributed among the centers. A descriptive analysis was performed, and results were compared to existing guidelines, as well as a previous survey in 2016. 79% of centers were adhering to the HARNESS‐MRI protocol in all epilepsy patients. All centers were acquiring 3D T1‐weighted sequences, 90% were acquiring 3D FLAIR and 87% were acquiring high in‐plane 2D coronal T2 MRI sequences in all epilepsy patients. In comparison, in 2016, only 50% of centers were following MRI recommendations at the time. Across European expert epilepsy centers, there has been increased harmonization of MRI sequences since the introduction of the HARNESS‐MRI protocol. This standardization supports optimal radiological review at individual centers as well as enabling harmonization of multicenter datasets for research.

**Plain Language Summary:**

Neuroimaging investigations are a fundamental component of epilepsy diagnosis. The International League Against Epilepsy (ILAE) has created guidelines about what MRI images to obtain in all epilepsy patients. In this study, we assessed the adherence of expert European epilepsy centers to these guidelines and found that 79% are acquiring the minimum set of MRI scans in all epilepsy patients. Standardization of MRI imaging serves to improve epilepsy diagnosis across Europe.

**Key Points**

- 79% of EpiCARE centres are adhering to the HARNESS‐MRI protocol in all epilepsy patients.
- The introduction of the ILAE HARNESS‐MRI protocol has increased harmonisation of structural MRI sequences across European expert epilepsy centres.
- Standardisation of epilepsy MRI protocols supports optimal radiological review and facilitates engagement in multi‐centre research.

## 1 INTRODUCTION

Neuroimaging is an essential component in the diagnosis of patients with epilepsy. In recent years, there have been tremendous advances in the availability and acquisition of structural magnetic resonance imaging (MRI), functional MRI, positron emission tomography (PET), and single‐photon emission computed tomography (SPECT) imaging. Furthermore, advanced postprocessing methods and machine‐learning algorithms have been applied to neuroimaging data in patients with epilepsy. In 2019, the International League Against Epilepsy (ILAE) published recommendations for the use of structural MRI in patients with epilepsy^1^ which included the harmonized neuroimaging of epilepsy structural sequences (HARNESS‐MRI) protocol. This protocol advocates for high contrast, 3D T1‐weighted, and fluid‐attenuated inversion recovery (FLAIR) sequences with isotropic voxels as well as high in‐plane resolution 2D coronal T2‐weighted MRI. By providing a protocol that is applicable to pediatric and adult patients, 1.5T or 3T MRI scanners, and generalizable to specialist epilepsy centers as well as general hospitals, the recommendations aimed to enable consistent use of structural MRI in epilepsy. Furthermore, it was suggested that combining the HARNESS‐MRI protocol with postprocessing methods could identify previously unseen lesions, converting patients from “MRI negative” to “MRI positive”. This may have a transformative impact on their care as the absence of a lesion on MRI has consistently been associated with higher likelihood of surgical failure.^2^, ^3^ Adherence by epilepsy centers to the ILAE recommendations serves to ensure a minimum data quality standard for all epilepsy patients, maximize the diagnostic yield from structural neuroimaging, support further use of MRI postprocessing methods, and promote engagement in multicenter research, where specific MRI sequences are required.

The European Reference Network (ERN) EpiCARE was launched in 2017, co‐funded by the European Union (EU), with the main aim of helping patients with rare and complex epilepsies. The consortium currently includes 38 full members and 12 affiliated partners across 26 countries within the EU. In 2014/15, a survey was conducted of 25 European epilepsy surgery centers^4^ and found 63% acquired 3D T1‐weighted sequences with isotropic voxels, 67% acquired 2D coronal FLAIR sequences with slice thicknesses <3 mm and 63% acquired 2D coronal T2 with slice thicknesses <3 mm. Of note 3D FLAIR with isotropic voxels was not surveyed as it did not form part of existing recommendations at the time.

The aim of this study was to survey the diagnostic imaging and postprocessing techniques currently used by EpiCARE centers and to establish whether there is harmonization in neuroimaging diagnosis across EU specialist epilepsy centers. In addition, by comparing results to the previous survey of European epilepsy centers,^4^ we aimed to establish whether the introduction of structural MRI recommendations has led to improved standardization in the neuroimaging of epilepsy patients in the EU.

## 2 MATERIALS AND METHODS

A survey on neuroimaging practices was created using Google Forms and distributed to EpiCARE centers. The survey questions included: which MRI sequences were part of the standard MRI epilepsy protocol in all patients, which additional MRI sequences are acquired in selected patient groups, what MRI postprocessing is available, and whether PET or SPECT is available. A copy of the survey questions is available in Data S1. Data were collected between March 2022 and March 2023 and analyzed using Microsoft Excel.

Data on the number of European epilepsy centers that in 2014/15 acquired 3D T1‐weighted sequences with isotropic voxels, 2D coronal FLAIR sequences with slice thicknesses <3 mm and 2D coronal T2 with slice thicknesses <3 mm were extracted from the publication of the survey in Epilepsia.^4^ To establish whether the introduction of structural MRI recommendations has led to an increased standardization within European epilepsy centers, the percentage of centers acquiring 3D T1, FLAIR, and high in‐plane resolution 2D coronal T2‐weighted MRI was compared between the two surveys. A Fisher exact test was used to assess whether the proportion of centers completely adhering to MRI recommendations has increased since the introduction of the HARNESS‐MRI protocol.

## 3 RESULTS

Seventy‐eight percent (39/50) of centers responded to the survey, 37 of which were epilepsy surgery centers. Ninety‐two percent (36/39) of centers have access to a 3T MRI scanner, with the remaining three centers having a 1.5T scanner only. Eighty‐two percent (32/39) have a 1.5T and 3T MRI scanner and five centers (13%) have a 7T scanner. All centers had sedation available and 95% (37/39) had facilities for general anesthesia for MRI.

### 3.1 Adherence to the HARNESS‐MRI protocol

**FIGURE 1:**
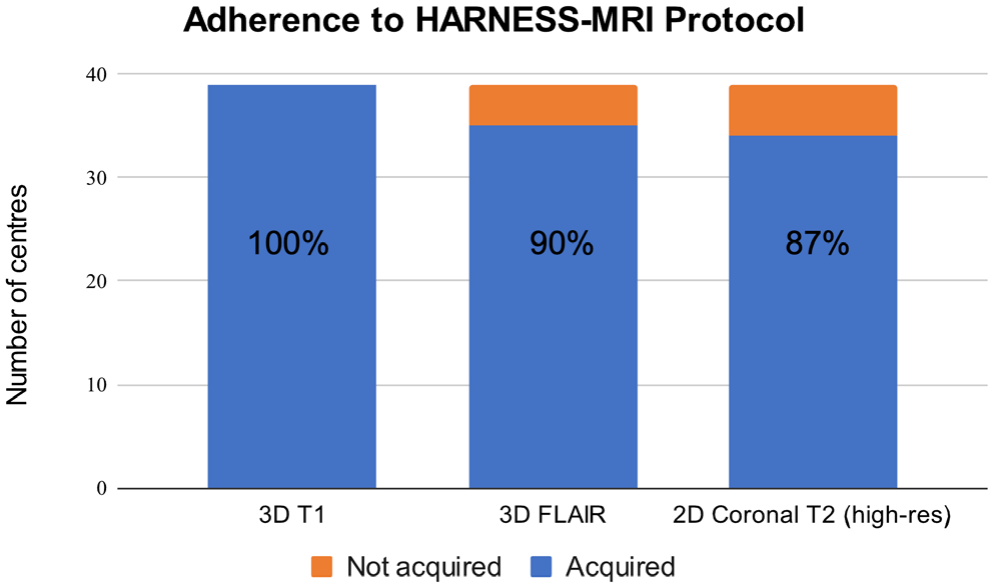
Number of centers that include HARNESS‐MRI guidelines recommended MRI sequences in their standard MRI protocol for all epilepsy patients.

All centers acquire 3D T1‐weighted MRI with isotropic voxels as part of their basic epilepsy protocol (Figure 1). Ninety percent (35/39) acquire 3D FLAIR with isotropic voxels as part of their basic epilepsy protocol (Figure 1) with the remaining four centers acquiring it in presurgical patients or patients with tumors, vascular malformations, or infectious processes. 87% (34/39) of centers acquire high in‐plane resolution 2D coronal T2‐weighted MRI (Figure 1). Of the remaining five centers, two centers do not perform high in‐plane resolution 2D coronal T2‐weighted MRI and three centers acquire it in presurgical patients or patients with tumors, vascular malformations, or infectious processes. Overall, 79% (31/39) of centers are adhering to the HARNESS‐MRI protocol as part of their basic epilepsy protocol (i.e. in all epilepsy patients and not only in select cohorts). This includes the two nonsurgical centers that responded to the survey.

Despite the expansion of the EpiCARE consortium from 25 centers to 50, since 2016, all centers are now acquiring 3D T1 in all epilepsy patients (a 37% increase). There is a 24% increase in the number of centers acquiring high in‐plane resolution 2D coronal T2‐weighted MRI. The acquisition of 3D FLAIR was not reported in the previous study,^4^ yet 67% of centers were acquiring either 2D or 3D FLAIR, indicating that there has been at least a 23% increase in the acquisition of 3D FLAIR. Lastly, in 2016, only 50% (12/24) of centers were following MRI recommendations at the time (isotropic 3D T1 and axial T2, coronal T2, axial FLAIR, and coronal FLAIR at less than 3 mm slice thickness). Thus, there has been a 29% increase (from 50% [12/24] to 79% [31/39]) in adherence to MRI recommendations (*p* = 0.012).

### 3.2 Additional MRI sequences

**FIGURE 2:**
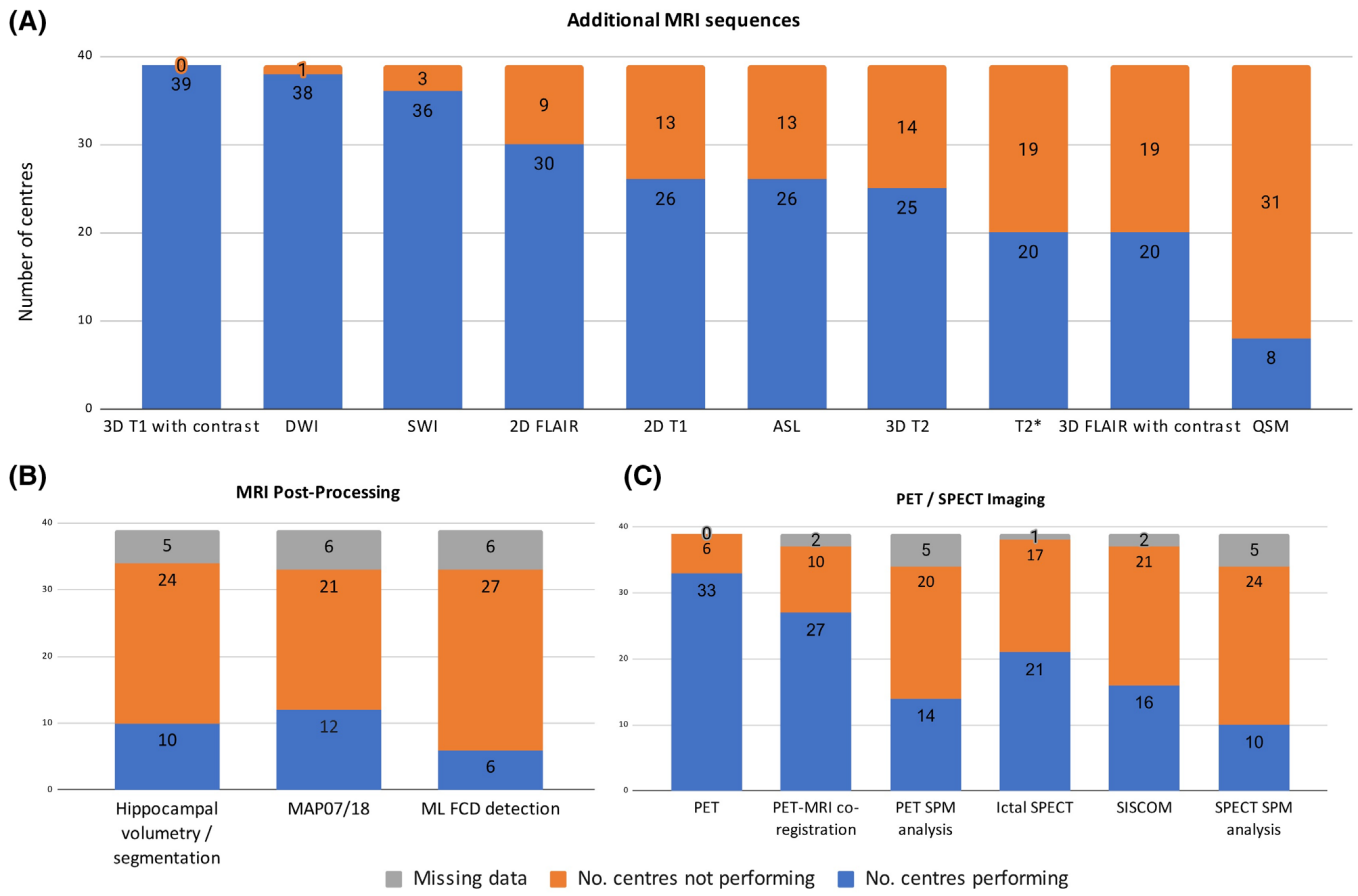
Additional imaging performed at EpiCARE centers. (A) Additional MRI sequences acquired in selected cohorts of patients. (B) MRI postprocessing availability. (C) PET and SPECT imaging and postprocessing availability. ASL, arterial spin labeling; DWI, diffusion‐weighted imaging; FCD, focal cortical dysplasia; FLAIR, fluid‐attenuated inversion recovery; MAP, morphometric analysis program; ML, machine‐learning; PET, positron emission tomography; QSM, quantitative susceptibility mapping; SPECT, single‐photon emission computed tomography; SPM, statistical parametric mapping; SWI, susceptibility‐weighted imaging.

All centers perform additional MRI sequences in selected cohorts such as presurgical patients or patients with tumors, vascular malformations, or infectious processes. In these selected cohorts, the most commonly acquired additional sequences were 3D T1 with contrast (100%, 39/39), diffusion‐weighted imaging (DWI) (97%, 38/39), and susceptibility‐weighted imaging (SWI) (92%; 36/39) (Figure 2A). Language functional MRI is performed at 87% (33/38) of centers, whereas memory fMRI is only performed at 41% (13/32). Seventy‐seven percent (30/39) of centers perform magnetic resonance spectroscopy (MRS) in selected cohorts. Where the number of centers is less than 39, this indicates the amount of missing data for this question of the survey.

The following additional MRI sequences are only acquired in small numbers of EpiCARE centers: Magnetization Prepared Two Rapid Acquisition Gradient Echo (MP2RAGE), fluid and white matter suppression (FLAWS), T2 relaxometry, T1 or T2 inversion recovery/double inversion recovery/Fast Gray Matter Acquisition T1 Inversion Recovery (FGATIR) sequences, Time‐of‐flight (TOF) magnetic resonance angiography (MRA), fast spin‐echo proton density‐weighted sequences as well as resting‐state functional MRI.

### 3.3 PET/SPECT imaging

Positron emission tomography (PET) imaging is utilized in selected epilepsy patients in 85% (33/39) of centers with 82% of centers that perform PET (27/33) co‐registering PET data to structural MRI imaging and 42% (14/33) using Statistical Parametric Mapping (SPM) analysis for PET postprocessing (Figure 2C). SPECT imaging is only performed in 55% (21/38) of EpiCARE centers (Figure 2C) with 49% (18/37) performing SPECT postprocessing (either SISCOM or SPECT SPM analysis).

### 3.4 MRI postprocessing

MAP‐07 or MAP18, the postprocessing software developed by Professor Huppertz^5^ to detect focal cortical dysplasias, is the most commonly used postprocessing technique with uptake by 36% (12/33) of EpiCARE centers (Figure 2B). 29% (10/34) of centers are performing hippocampal volumetry or hippocampal segmentations to assist with the detection of hippocampal sclerosis. Eighteen percent (6/33) of centers are using openly available machine‐learning algorithms^6^, ^7^ to assist with the detection of focal cortical dysplasias. Where the number of centers is less than 39, this indicates the amount of missing data for this question of the survey.

## 4 DISCUSSION

This survey on the use of neuroimaging in 39 EpiCARE centers demonstrates that since the introduction of the ILAE HARNESS‐MRI protocol^1^ harmonization of MRI protocols across expert epilepsy centers has increased, with 79% of centers adhering to the recommendations for their basic epilepsy protocol. However, there is still wide variability among centers in terms of additional MRI sequences performed, and the use of PET and SPECT imaging and MRI postprocessing techniques.

The increase in standardization of MRI protocols has a number of important potential implications. First, the protocol was designed so that the 3D T1 optimally evaluates brain anatomy and morphology, the 3D FLAIR is particularly beneficial for hyperintense cortical lesions, and the high in‐plane 2D coronal T2 MRI sequences to well visualize the internal hippocampal structure.^1^ There is evidence that the use of dedicated epilepsy MRI protocols alongside evaluation by experienced epilepsy neuroradiologists improves lesion detection.^8^, ^9^ As such, adherence to the protocol serves to maximize the diagnostic yield of clinical neuroimaging. Second, a particular strength of EpiCARE is the surgical and nonsurgical case discussions where a medical team from one center presents a challenging case to expert epileptologists, neuroradiologists, neurophysiologists, neurosurgeons, and epilepsy specialist nurses from across the EpiCARE network. Harnessing expertise from across the EU, a plan for the patient's ongoing care is discussed. The MRI imaging for the patient is a crucial investigation that is presented and standardization across centers enables a common framework for the discussion of rare and complex patients. Third, consistent and reliable imaging data across different imaging centers reduces the variability in image acquisition and can facilitate the sharing of data between different centers and research groups, which can enhance collaboration and support participation in multicenter neuroimaging research studies such as ENIGMA epilepsy^10^, ^11^ and the MELD Project.^7^, ^12^

However, the ILAE HARNESS‐MRI protocol was intended as a minimum set of MRI basic sequences that could be performed on all epilepsy patients regardless of setting, that is, in general hospitals as well as epilepsy surgery centers. Despite this, 21% of specialist epilepsy centers in the EU are not currently performing isotropic, millimetric 3D T1 and FLAIR images, and high‐resolution 2D submillimetric T2 images in all epilepsy patients. Although we do not have data on the reasons why certain centers do not follow the ILAE HARNESS‐MRI protocol, we hypothesize that this is due to lack of awareness of the current guidelines, lack of time or expertise to change current MRI protocols, preference by individual radiologists for high in‐plane resolution 2D FLAIR images over 3D images or the use of MRI scanners that are not optimized for high‐quality 3D FLAIR. Furthermore, beyond expert epilepsy centers, we do not have data on the uptake of the HARNESS‐MRI protocol in less specialist settings. Further work is needed to assess and increase the uptake of the HARNESS‐MRI in all settings.

All centers are acquiring additional MRI sequences in their epilepsy patients. Given that 92% of centers are acquiring DWI and 79% are acquiring SWI as part of their basic epilepsy protocol, guidelines on the acquisition of DWI and SWI would be beneficial to standardize these imaging modalities.

The ILAE Neuroimaging task force endorses the use of neuroimaging postprocessing^1^ to help characterize pathology. Within the EpiCARE network, 56% of centers are performing hippocampal volumetry or segmentation, using postprocessing methods to assist with the detection of focal cortical dysplasias or using in‐house structural MRI postprocessing tools. This is encouraging, but education and training are needed to support other centers with the technical skills to perform structural MRI postprocessing, and evaluation studies are required to determine the added benefit of these postprocessing tools in the diagnostic workup of patients with complex epilepsy.

There are a number of limitations associated with this study. First, it is important to note that only 78% of EpiCARE centers responded to the survey. Second, the survey questions were not identical to the survey of European Epilepsy Centres published in 2016.^4^ Last, as data from the Mouthaan et al., study was extracted from the published manuscript, it was not possible to assess whether there were changes or improvements within individual centers that contributed to both surveys. Rather, both surveys provide a snapshot overview of what MRI sequences are being acquired across expert European epilepsy centers.

In conclusion, the improvement in standardization in the basic epilepsy MRI protocol from 50% to 79% of EpiCARE centers correlates with the introduction of the ILAE Neuroimaging Task Force recommendation of the HARNESS‐MRI protocol. This supports the important role of establishing protocols for MRI acquisition in epilepsy. However, the variability in terms of additional MRI sequences, acquisition of PET or SPECT imaging, and the use of postprocessing techniques suggests an important role for further guidelines regarding these additional neuroimaging modalities and techniques. Furthermore, further education and training are required to increase the uptake of the HARNESS‐MRI protocol across specialist epilepsy centers and beyond and to train clinicians and researchers in the use of neuroimaging postprocessing techniques.

## CONFLICT OF INTEREST STATEMENT

None of the authors have any conflicts of interest to disclose. We confirm that we have read the Journal's position on issues involved in ethical publication and affirm that this report is consistent with those guidelines.

## Supporting information


Data S1.

## Data Availability

The data that support the findings of this study are available from the corresponding author upon reasonable request.
