# Supplementary material for: Harmonization of MRI sequences across ERN EpiCARE centers
Source: Epilepsia Open. 2025 Feb 12;10(2):587–92. doi: 10.1002/epi4.13115 (PMC12014919; doi:10.1002/epi4.13115)
Supplement: Supplementary file 1 — Data S1. [file EPI4-10-587-s001.docx]

**Harmonisation of MRI sequences across ERN EpiCARE centres**

**Supplementary Material**

**List of survey questions:**

1. What is the name of EpiCARE Centre you are based at?

2. In which country is the EpiCARE centre?

3. Does your centre perform epilepsy surgery?

- - Yes - in adults only
  - Yes - in children only
  - Yes - in adults and children
  - No

4. Which field strength MRI scanners are in use at your centre? (Tick all that apply)

- 1.5T
- 3T
- 7T

5. Do you have facilities for general anaesthesia or sedation for MRI?

|  | Yes - in adults | Yes - in children | Not Available |
| --- | --- | --- | --- |
| General Anaesthesia |  |  |  |
| Sedation |  |  |  |

6. For clinical purposes, do you acquire the following sequences in your epilepsy patients? (Tick all that apply)

|  | All epilepsy patients (Basic protocol) | Presurgical patients | Tumours/ Vascular malformations / Infectious processes | Tuberous sclerosis | Neonates | Other subgroups | Not performed |
| --- | --- | --- | --- | --- | --- | --- | --- |
| 3D T1 |  |  |  |  |  |  |  |
| 3D FLAIR |  |  |  |  |  |  |  |
| 2D coronal T2 (high-res) |  |  |  |  |  |  |  |
| 3D T2 |  |  |  |  |  |  |  |
| 3D T1 with contrast |  |  |  |  |  |  |  |
| 3D FLAIR with contrast |  |  |  |  |  |  |  |
| 2D T1 |  |  |  |  |  |  |  |
| 2D FLAIR |  |  |  |  |  |  |  |
| DWI / ADC |  |  |  |  |  |  |  |
| SWI |  |  |  |  |  |  |  |
| T2* |  |  |  |  |  |  |  |
| ASL |  |  |  |  |  |  |  |
| QSM (Quantitative susceptibility mapping) |  |  |  |  |  |  |  |

7. If you ticked "Other subgroups" for particular sequences, please detail which groups of patients you acquire them in.

8. For clinical purposes, do you acquire any other MRI sequences (not detailed earlier in the survey)? Please provide details of the sequence and the groups of patients you acquire them in.

9. For clinical purposes, do you perform any of the following imaging?

|  | Yes | No |
| --- | --- | --- |
| Ictal SPECT |  |  |
| SISCOM |  |  |
| SPM SPECT |  |  |
| PET |  |  |
| PET - MRI co-registration |  |  |
| PET SPM analysis |  |  |
| DTI tractography |  |  |
| Language fMRI |  |  |
| Memory fMRI |  |  |
| MR Spectroscopy |  |  |
| Hippocampal volumetry / segmentation |  |  |
| T2 relaxometry |  |  |
| MAP-07 / MAP-18 |  |  |
| Fully automated lesion detection techniques (e.g. Gill et al., 2021, MELD project) |  |  |

10. For clinical purposes, do you perform any other imaging techniques / postprocessing (not detailed earlier in the survey)? Please provide details of the technique and the groups of patients that you perform it on.

11. If you incorporate any post-processing technique into patient care, please provide details of how (e.g. in a clinical trial, presented at the multi-disciplinary team meeting, used by the radiologist only, incorporated into surgical planning software e.g. EpiNav, not incorporated into clinical care)
